# Examining the association between serum phosphate levels and leukocyte telomere length

**DOI:** 10.1038/s41598-020-62359-3

**Published:** 2020-03-25

**Authors:** Zhe-Yu Yang, Tung-Wei Kao, Tao-Chun Peng, Yuan-Yuei Chen, Hui-Fang Yang, Chen-Jung Wu, Wei-Liang Chen

**Affiliations:** 1 Department of General Medicine, Tri-Service General Hospital, and School of Medicine, National Defense Medical Center, Taipei, Taiwan, Republic of China; 2 Division of Family Medicine, Department of Family and Community Medicine, Tri-Service General Hospital, and School of Medicine, National Defense Medical Center, Taipei, Taiwan, Republic of China; 3 Division of Geriatric Medicine, Department of Family and Community Medicine, Tri-Service General Hospital, and School of Medicine, National Defense Medical Center, Taipei, Taiwan, Republic of China; 4 Graduate Institute of Clinical Medical, College of Medicine, National Taiwan University, Taipei, Taiwan, Republic of China; 5 Department of Pathology, Tri-Service General Hospital, and School of Medicine, National Defense Medical Center, Taipei, Taiwan, Republic of China; 6 Division of Family Medicine, Department of Community Medicine, Taoyuan Armed Forces General Hospital, Taoyuan, Taiwan, Republic of China

**Keywords:** Prognostic markers, Health occupations

## Abstract

Accelerated telomere attrition is related to various diseases, and multiple factors have been reported to influence telomere length. However, little attention has focused on the relationship between serum phosphate levels and mean telomere length. The purpose of this study was to explore the relationship between serum phosphate levels and mean telomere length in the US general population. A total of 7,817 participants from the 1999–2002 NHANES were included. The association between serum phosphate levels and mean telomere length was investigated using regression models. A remarkably positive relationship between serum phosphate levels and mean telomere length emerged after adjustments were made for covariates. The adjusted β coefficient of serum phosphate levels for mean telomere length was 0.038 (95% confidence intervals (CIs), 0.022 to 0.095, p = 0.002). A longer telomere length was observed in participants with serum phosphate levels in the highest quartiles, and a dose-dependent association was observed. Our study demonstrated that higher quartiles of phosphate had a remarkable correlation with longer telomere length.

## Introduction

Phosphate is an essential mineral in the body^1^ and plays a crucial role in many physiological processes, such as energy generation, signal transduction, acid-base balance and bone mineralization^2^. Phosphate homeostasis is modulated by dietary phosphate intake, intestinal absorption, renal reabsorption and excretion^3^. There are various regulators of phosphate metabolism, including parathyroid hormone (PTH), calcitriol, vitamin D and fibroblast growth factor 23 (FGF23)^2^. And imbalance of phosphate homeostasis may induce hypo- and hyperphosphatemia^4^. Hyperphosphatemia is correlated with a higher risk of cardiovascular diseases^5^ and all-cause mortality^6^. In contrast, hypophosphatemia is associated with hypertension^7^ and reduced insulin resistance^8^. Kalaitzidis, R. *et al*. reported that individuals with metabolic syndrome had remarkably lower serum phosphate levels than did healthy participants^9^. Hence, higher and lower serum phosphate levels have been associated with, respectively, increased cardiovascular risks and more parameters of metabolic syndrome.

Telomeres capping the end of eukaryotic chromosomes protect chromosomes from loss and end-to-end fusion^10^. Telomeres shorten with repeated cell division and DNA replication^11^. Telomere length has been positively correlated with healthy life years in elderly individuals^12^. Furthermore, shorter telomere length is correlated with higher all-cause mortality^13^. Numerous factors have been found to be associated with telomere length^14^. Accelerated telomere attrition was correlated with lower socioeconomic status and poor diet^15^. A review in 2011 demonstrated that various nutrients, such as vitamin A, vitamin D, folate, vitamin B12, magnesium, zinc and iron, may affect telomere length^16^. In a cross-sectional analysis, Xu, Q., *et al*. found a positive relationship between multivitamin use and telomere length in women^17^. Higher dietary intake of magnesium was also associated with longer telomere length^17^. In contrast, shorter telomere length was found in iron supplement users^18^.

Despite a large amount of evidence on factors affecting telomere length, little research has clarified the relationship between serum phosphate and telomere length. The aim of our study was to investigate the correlation between serum phosphate and mean telomere length in the US general population.

## Results

### Characteristics of the study population

The characteristics of the participants divided by serum phosphate quartiles are listed in Table 1. In the study, the mean age of all participants was 49.42 ± 18.82 years, and 48.2% of all participants were men. Mean telomere length, creatinine and total calcium levels were significantly positively associated with serum phosphate levels, whereas age, body mass index (BMI), SBP, serum FG and serum albumin were negatively correlated with serum phosphate levels.

**Table 1:** Characteristics of Study Participants of Quartiles of serum phosphate.

Characteristics of Study Participants	Quartiles of serum phosphate					
	Q1 (0.55–1.033)	Q2 (1.033–1.16)	Q3 (1.16–1.26)	Q4 (1.26–2.39)	Total	*P* Value
	(n = 2030)	(n = 2018)	(n = 1869)	(n = 1900)	(n = 7817)	
**Continuous variables**^**a**^						
Mean telomere length (T/S ratio)	0.99 (0.31)	1.01 (0.25)	1.04 (0.26)	1.06 (0.26)	1.02 (0.28)	<0.001
Age (years)	52.61 (18.0)	50.51 (18.78)	48.65 (19.01)	45.62 (18.81)	49.42 (18.82)	<0.001
BMI (kg/m^2^)	28.81 (6.14)	28.36 (6.20)	28.07 (6.04)	28.03 (6.20)	28.33 (6.15)	<0.001
SBP (mm-Hg)	130.07 (21.92)	128.31 (22.65)	126.79 (22.49)	124.21 (21.25)	127.42 (22.19)	<0.001
Serum FG (mg/dL)	102.24 (39.43)	96.28 (30.52)	96.18 (35.88)	94.41 (34.25)	97.35 (35.29)	<0.001
Serum TC (mg/dL)	199.23 (40.79)	198.54 (38.64)	201.86 (42.93)	202.90 (42.48)	200.57 (41.22)	0.002
Serum albumin (g/dL)	4.32 (0.34)	4.30 (0.34)	4.30 (0.35)	4.25 (0.39)	4.30 (0.36)	<0.001
ALT (U/L)	26.13 (21.61)	24.72 (18.19)	25.59 (35.42)	26.80 (48.32)	25.80 (32.78)	0.238
Creatinine (mg/dL)	0.80 (0.27)	0.83 (0.39)	0.80 (0.42)	0.90 (0.76)	0.83 (0.49)	<0.001
Total calcium (mg/dL)	9.31 (0.42)	9.38 (0.39)	9.42 (0.38)	9.47 (0.41)	9.39 (0.41)	<0.001
Serum CRP (mg/dL)	0.53 (1.17)	0.47 (0.89)	0.46 (0.77)	0.48 (0.81)	0.49 (0.93)	0.065
**Categorical variables**^**b**^						
Male	1147 (57.8)	1013 (50.2)	812 (43.4)	767 (40.4)	3766 (48.2)	<0.001
Non-Hispanic white	1018 (50.1)	1057 (52.4)	941 (50.3)	943 (49.6)	3959 (50.6)	0.188
Congestive heart failure	71 (3.5)	54 (2.7)	56 (3.0)	48 (2.5)	229 (2.9)	0.292
Coronary heart disease	105 (5.2)	83 (4.1)	73 (3.9)	67 (3.5)	328 (4.2)	0.335
Angina/angina pectoris	82 (4.0)	76 (3.8)	67 (3.6)	53 (2.8)	278 (3.6)	0.234
Heart attack	99 (4.9)	83 (4.1)	86 (4.6)	67 (3.5)	335 (4.3)	0.278
Stroke	71 (3.5)	59 (2.9)	49 (2.6)	58 (3.1)	237 (3.0)	0.721
Cancer or malignancy	194 (9.6)	173 (8.6)	146 (7.8)	131 (6.9)	644 (8.2)	0.070
Smoking	993 (48.9)	973 (48.3)	865 (46.3)	961 (50.6)	3792 (48.5)	0.254

Abbreviation:

BMI, body mass index; SBP, systolic blood pressure; Serum FG, serum fasting glucose; Serum TC, serum total cholesterol; ALT, alanine; CRP, C-reactive protein.

^a^Values were expressed as mean (standard deviation).

^b^Values in the categorical variables were expressed as number (%).

### Association between serum phosphate levels and mean telomere length after stratification by sex and ethnicity

Tables 2, 3 presents the results of the correlation between serum phosphate levels and mean telomere length stratified by sex and ethnicity. A significant correlation was found between higher serum phosphate levels and longer mean telomere lengths in the three models (p < 0.05). Additionally, male participants demonstrated an association between higher serum phosphate levels and longer mean telomere lengths in model 2 and in the fully adjusted model (model 2: β = 0.043, p = 0.011; model 3: β = 0.045, p = 0.009). In the non-Hispanic white group and other racial groups, a positive association between serum phosphate levels and mean telomere length was found in 3 adjusted models (p < 0.05).

**Table 2:** Gender-specific association between the mean Telomere Length and the serum phosphate level.

		Model^a^ 1		Model^a^ 2		Model^a^ 3	
		β^b^ (95% CI)	*P* Value	β^b^ (95% CI)	*P* Value	β^b^ (95% CI)	*P* Value
serum phosphate level(mmol/L)	Total	0.034 (0.018, 0.087)	0.003	0.037 (0.020, 0.093)	0.002	0.038 (0.022, 0.095)	0.002
	Male	0.032 (0.000–0.103)	0.05	0.043 (0.016–0.123)	0.011	0.045 (0.018–0.126)	0.009
	Female	0.031 (0.001–0.093)	0.044	0.028 (−0.006–0.091)	0.088	0.030 (−0.005–0.093)	0.077

^a^Adjusted covariates:

Model 1 = age, sex, race/ethnicity,

Model 2 = Model 1 + BMI, systolic blood pressure, serum fasting glucose, serum total cholesterol, serum albumin, ALT, total calcium, serum C-reactive protein, creatinine.

Model 3 = Model 2 + history of congestive heart failure, coronary heart disease, angina/angina pectoris, heart attack, stroke, cancer/malignancy and smoking.

^b^β coefficients was interpreted as change of telomere length for each increase in serum phosphate.

**Table 3:** Ethnicity specific association between the mean Telomere Length and the serum phosphate level.

		Model^a^ 1		Model^a^ 2		Model^a^ 3	
		β^b^ (95% CI)	*P* Value	β^b^ (95% CI)	*P* Value	β^b^ (95% CI)	*P* Value
serum phosphate level(mmol/L)	Total	0.034 (0.018, 0.087)	0.003	0.037 (0.020, 0.093)	0.002	0.038 (0.022, 0.095)	0.002
	Mexican American	0.051 (0.007–0.171)	0.034	0.034 (−0.029–0.147)	0.186	0.033 (−0.031–0.146)	0.205
	Other Hispanic	0.038 (−0.084–0.191)	0.447	0.030 (−0.110–0.192)	0.592	0.035 (−0.100–0.199)	0.518
	Non-Hispanic White	0.034 (0.005–0.095)	0.031	0.040 (0.012–0.107)	0.014	0.041 (0.013–0.108)	0.013
	Non-Hispanic Black	−0.011 (−0.100–0.067)	0.695	−0.003 (−0.092–0.084)	0.925	−0.002 (−0.092–0.087)	0.952
	Other Race	0.135 (0.008–0.331)	0.039	0.150 (0.023–0.356)	0.026	0.145 (0.012–0.354)	0.037

^a^Adjusted covariates:

Model 1 = age, sex, race/ethnicity,

Model 2 = Model 1 + BMI, systolic blood pressure, serum fasting glucose, serum total cholesterol, serum albumin, ALT, total calcium, serum C-reactive protein, creatinine.

Model 3 = Model 2 + history of congestive heart failure, coronary heart disease, angina/angina pectoris, heart attack, stroke, cancer/malignancy and smoking.

^b^β coefficients was interpreted as change of telomere length for each increase in serum phosphate.

After serum phosphate levels were stratified into four quartiles, gender-specific associations between serum phosphate levels and mean telomere length were determined and are listed in Table 4. Table 4 shows a significantly positive association between the highest serum phosphate level (Q4) and mean telomere length for all designed models (p < 0.05). The mean telomere length was greater in the higher quartiles than in the lowest quartile of serum phosphate, and a dose-dependent association was observed. Male participants with the highest serum phosphate levels (Q4) had a longer mean telomere length than did those with serum phosphate levels in Q1 in the 3 adjusted models.

**Table 4:** Gender-specific association between the mean Telomere Length and the serum phosphate level.

Models^a^	Quartiles	Total		Male		Female	
		β^b^ (95% CI)	*P* Value	β^b^ (95% CI)	*P* Value	β^b^ (95% CI)	*P* Value
Model 1	Q2 v.s. Q1 Q3 v.s. Q1 Q4 v.s. Q1	0.013 (−0.009,0.025) 0.035 (0.006, 0.041) 0.038 (0.008, 0.043)	0.345 0.009 0.005	0.01 (−0.018–0.031) 0.028 (−0.006–0.047) 0.037 (0.001–0.054)	0.601 0.125 0.045	0.013 (−0.016–0.031) 0.038 (−0.001–0.046) 0.036 (−0.002–0.044)	0.522 0.057 0.078
Model 2	Q2 v.s. Q1 Q3 v.s. Q1 Q4 v.s. Q1	0.010 (−0.010, 0.024) 0.033 (0.004, 0.040) 0.039 (0.007, 0.043)	0.442 0.015 0.006	0.010 (−0.018–0.032) 0.030 (−0.004–0.049) 0.047 (0.007–0.062)	0.577 0.102 0.015	0.009 (−0.019–0.029) 0.034 (−0.004–0.044) 0.031 (−0.006–0.042)	0.666 0.098 0.138
Model 3	Q2 v.s. Q1 Q3 v.s. Q1 Q4 v.s. Q1	0.010 (−0.011, 0.023) 0.033 (0.004, 0.040) 0.040 (0.008, 0.044)	0.473 0.015 0.005	0.010 (−0.018–0.032) 0.031 (−0.004–0.049) 0.049 (0.008–0.063)	0.582 0.098 0.011	0.008 (−0.019–0.028) 0.033 (−0.004–0.044) 0.032 (−0.005–0.043)	0.703 0.101 0.123

^a^Adjusted covariates:

Model 1 = age, sex, race/ethnicity,

Model 2 = Model 1 + BMI, systolic blood pressure, serum fasting glucose, serum total cholesterol, serum albumin, ALT, total calcium, serum C-reactive protein, creatinine

Model 3 = Model 2 + history of congestive heart failure, coronary heart disease, angina/angina pectoris, heart attack, stroke, cancer/malignancy and smoking

^b^β coefficients was interpreted as change of telomere length for each increase in serum phosphate.

Table 5 presents the ethnicity-specific association between serum phosphate levels and mean telomere length after multivariable logistic regression analysis. Only in the non-Hispanic white group, participants with higher quartiles of serum phosphate levels (Q2, Q3 and Q4) had a longer mean telomere length than did participants with serum phosphate levels in Q1 in the 3 regression models (p < 0.05).

**Table 5:** Ethnicity specific association between the mean Telomere Length and the serum phosphate level.

Models^a^	Quartiles	Mexican American		Other Hispanic		Non-Hispanic White		Non-Hispanic Black		Other Race	
		β^b^ (95% CI)	*P* Value	β^b^ (95% CI)	*P* Value	β^b^ (95% CI)	*P* Value	β^b^ (95% CI)	*P* Value	β^b^ (95% CI)	*P* Value
Model 1	Q2 v.s. Q1 Q3 v.s. Q1 Q4 v.s. Q1	0.015 (−0.029–0.050) 0.003 (−0.038–0.042) 0.047 (−0.006–0.075)	0.601 0.918 0.095	0.037 (−0.111–0.035) 0.038 (−0.053–0.096) 0.036 (−0.047–0.095)	0.308 0.563 0.503	0.052 (0.009–0.053) 0.062 (0.016–0.061) 0.052 (0.010–0.056)	0.005 0.001 0.005	−0.084 (−0.098–0.010) −0.013 (−0.054–0.036) −0.042 (−0.071–0.018)	0.016 0.697 0.236	−0.070 (−0.124–0.044) 0.120 (−0.016–0.159) 0.140 (−0.007–0.157)	0.352 0.111 0.073
Model 2	Q2 v.s. Q1 Q3 v.s. Q1 Q4 v.s. Q1	−0.001 (−0.041–0.039) −0.009 (−0.047–0.034) 0.026 (−0.024–0.061)	0.966 0.743 0.392	−0.046 (−0.103–0.047) 0.023 (−0.063–0.091) 0.039 (−0.053–0.099)	0.469 0.716 0.548	0.052 (0.009–0.053) 0.064 (0.017–0.063) 0.057 (0.012–0.059)	0.005 0.001 0.003	−0.093 (−0.105–0.015) −0.022 (−0.060–0.031) −0.044 (−0.074–0.017)	0.009 0.534 0.223	−0.066 (−0.125–0.050) 0.140 (−0.007–0.174) 0.159 (0.000–0.170)	0.402 0.070 0.049
Model 3	Q2 v.s. Q1 Q3 v.s. Q1 Q4 v.s. Q1	−0.001 (−0.041–0.039) −0.010 (−0.047–0.033) 0.024 (−0.025–0.060)	0.960 0.737 0.427	−0.022 (−0.089–0.063) 0.027 (−0.060–0.093) 0.062 (−0.040–0.112)	0.732 0.667 0.350	0.05 (0.008–0.052) 0.063 (0.017–0.062) 0.057 (0.012–0.059)	0.008 0.001 0.003	−0.092 (−0.104–0.014) −0.022 (−0.060–0.031) −0.043 (−0.074–0.018)	0.010 0.537 0.237	−0.065 (−0.128–0.054) 0.118 (−0.026–0.166) 0.156 (−0.004–0.171)	0.424 0.153 0.060

^a^Adjusted covariates:

Model 1 = age, sex, race/ethnicity,

Model 2 = Model 1 + BMI, systolic blood pressure, serum fasting glucose, serum total cholesterol, serum albumin, ALT, total calcium, serum C-reactive protein, creatinine.

Model 3 = Model 2 + history of congestive heart failure, coronary heart disease, angina/angina pectoris, heart attack, stroke, cancer/malignancy and smoking.

^b^β coefficients was interpreted as change of telomere length for each increase in serum phosphate.

## Discussion

In the present cohort study of the US general population, a positive relationship between serum phosphate levels and mean telomere length was observed. These findings are not consistent with those of previous studies^5,19–23^. Higher serum phosphate levels were noted to be linked with increased cardiovascular risk and mortality risk^5,19–21,23,24^. Furthermore, McClelland, R., *et al*. demonstrated that hyperphosphatemia was associated with accelerated aging, which was evaluated by markers of biological age (i.e., telomere length and DNA methylation content)^22^. Recently, emerging evidence on klotho expression and aging has been published^25–28^. Klotho was associated with phosphate homeostasis via the fibroblast growth factor (FGF) receptor, and decreased klotho expression may induce hyperphosphatemia^29^. Thus, a plausible association between phosphate toxicity and accelerated aging in klotho-deficient mice was also reported^30,31^.

Telomere length was viewed as a biomarker of age^32^. Accelerated telomere attrition was associated with various diseases, such as coronary heart disease^33^, diabetes mellitus^34^, hypertension^35^ and cancer^36^. Telomere length was influenced by multiple factors, including psychosocial, environmental, and behavioral factors^14^. Different lifestyles and diets also played prominent roles in telomere length^37,38^. Mirabello, L., *et al*. demonstrated that a healthy lifestyle with more exercise, cigarette abstinence and a diet high in fruit and vegetables were significantly correlated with longer telomere length^38^. Cassidy, A., *et al*. reported that intake of dietary fiber was positively linked with telomere length, while waist circumference, body mass index (BMI) and the intake of polyunsaturated fatty acids were negatively linked^37^. The inverse correlation between obesity and telomere length was observed in females^39^. Increased adiposity and increasing BMI were correlated with shorter telomere length^40^. A previous study by Song *et al*. revealed that BMI was significantly positively associated with biomarkers of DNA damage^41^. Furukawa *et al*. proposed that elevated chronic oxidative stress was observed in accumulated fat^42^. Kurz, D.J., *et al*. demonstrated that oxidative stress may accelerate telomere erosion^43^. Hence, we speculated that a higher BMI may increase oxidative stress, which could cause DNA damage and might induce telomere attrition.

Few studies have demonstrated a negative association between serum phosphate and BMI^44,45^. Lower BMI might increase the risk of hyperphosphatemia^46^. Haglin *et al*. observed that hypophosphatemia was correlated with higher BMI in females^47^. Our results were in agreement with these articles that showed an inverse relationship between serum phosphate and BMI. Several possible mechanisms have been proposed for this phenomenon. Haglin *et al*. speculated that it might be due to a high caloric diet with low nutrient density and low protein intake, which could cause phosphate depletion^47^. Obeid, O.A. hypothesized that lower serum phosphate may reduce ATP production, which is important for energy expenditure^45^. Depending on the inverse relationship, we hypothesized that a higher serum phosphate level might be associated with a lower BMI and that a lower BMI may be associated with a greater telomere length. Therefore, these hypothesized relationships might serve as a possible explanation for our results. The hypotheses that participants with higher serum phosphate levels had longer telomere lengths are biologically plausible.

Our study demonstrated that statistically significant relationships between higher serum phosphate levels and longer mean telomere length, particularly in male participants. Previous published studies have shown gender differences in serum phosphate levels^48,49^. Few studies have examined the effects of gonadal steroids on phosphorus homeostasis. Sex hormone deprivation was found to be correlated with elevated serum phosphate levels in men^50^. Moreover, testosterone and estrogen were associated with mean telomere length^51,52^. A possible mechanism may account for this phenomenon^53^.

There are several limitations in the present study. First, because our study was a cross-sectional observational analysis of a database, causal inferences were not clear. Further longitudinal studies are warranted. Second, adjustments were not made in regression models for unmeasured confounding variables, such as lifestyle factors and dietary patterns that might interfere with both mean telomere length and serum phosphate levels. Third, there might be a possible selection bias in our study. Our participants were mostly Caucasian; therefore, the generalizability of the results might be limited to specific racial populations.

In conclusion, a significant positive correlation between serum phosphate levels and mean telomere length was observed in the study. The findings of the paucity of currently published studies were consistent with our results, and our study provided epidemiologic evidence for further studies on the relationship between mean telomere length and serum phosphate levels.

## Materials and methods

### Study populations

All data were obtained from the National Health and Nutrition Examination Survey (NHANES) from 1999 to 2002. NHANES was a cross-sectional study of noninstitutionalized US citizens and was conducted by the Centers for Disease Control and Prevention (CDC) and the National Center for Health Statistics (NCHS). NHANES database included personal data including demographic information, past medical history and laboratory data. The participants’ information was collected by a household interview and a subsequent physical examination. All of the study protocol, consent documents and relevant information were detailed on the NHANES website. The NHANES study protocol was conducted according to the NCHS Institutional Review Board (IRB). Before data collection and the health examinations, all informed consents had been obtained. All the experimental protocols were approved by NCHS IRB. Initially, a total of 7,817 participants who aged 20 years old or older were included in our study.

### Covariates

**Figure 1:**
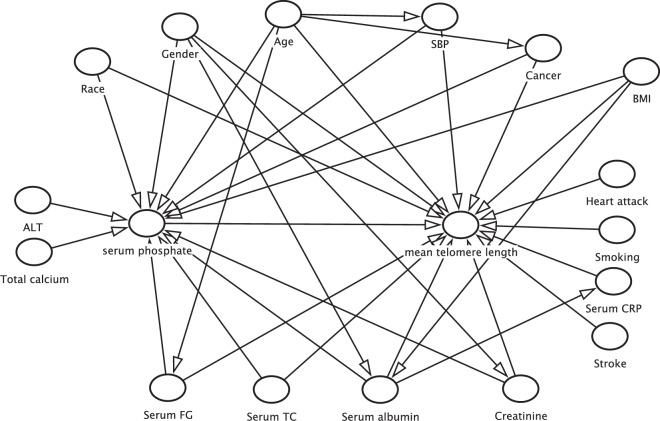
Directed Acyclic Graph of our study.

For causal relationship from cross-sectional study, it was essential to control confounding, but it was hard to recognize a potential confounder. A confounding variable was associated with the exposure and with the outcome or the occurrence of a disease. A confounder obscured the real causal path between the exposure and outcome. Based on the variables correlation and previous studies, we drew our Directed Acyclic Graph (DAG) analysis of the study, showed in Fig. 1. Demographic confounders were collected with a computer-assisted personal interviewing system, including age, gender, race, personal history, and past medical status. Other medical histories were recorded, including congestive heart failure, coronary artery disease, angina, stroke and cancer/malignancy, which were diagnosed or revealed by a doctor.

The level of serum C-reactive protein (CRP) was measured by the Dade Behring Nephelometer II Analyzer System using latex-enhance nephelometry (Dade Behring Diagnostics Inc., Somerville, NJ). The level of serum fasting glucose (FG) was measured by the Instrumentation Cobas Mira Chemistry System (Roche Diagnostic Systems, Inc., Montclair, New Jersey). The resting biochemical profiles, including creatinine, alanine aminotransferase (ALT), total calcium, serum total cholesterol (TC) and serum albumin, were measured with the Beckman Synchron LX20. All protocols followed the standardized guidelines and record accuracy based on CDC reference methods.

### Serum phosphate measurement

The level of serum phosphate was measured by a Hitachi model 737 multichannel analyzer (Boehringer Mannheim Diagnostics, Indianapolis, IN). Inorganic phosphorus reacted with ammonium molybdate in an acidic solution to make a colored phosphomolybdate complex. The quantification of serum phosphate was performed using the change in absorbance at 365 nm of phosphomolybdate.

### Mean telomere length measurement

The telomere length assay for the measurement of telomere length relative to standard reference DNA (T/S ratio) was performed using the quantitative polymerase chain reaction (PCR) method in the laboratory of Dr. Elizabeth Blackburn at the University of California, San Francisco. More detailed information can be found on the NHANES web site in the laboratory section.

### Statistical analysis

We used SPSS version 18 (SPSS Inc., Chicago, IL, USA) to perform all statistical analyses. Continuous variables are indicated as the means and standard deviations (SDs); categorical variables are indicated as numbers and percentages. The chi-square test and one-way ANOVA were used for categorical data and continuous data, respectively. To examine the variables of interest, we divided 7,817 participants into quartiles based on serum phosphate levels. Two-sided p-values <0.05 were considered statistically significant.

We investigated the relationship between serum phosphate levels and mean telomere length using multivariable logistic regression analysis. Covariate adjustments were conducted using 3 extended-model methods: model 1 was adjusted for age, gender and race; model 2 was further adjusted for associated clinical laboratory data; and model 3 was further adjusted for smoking history and past medical history. We tested for effect modification by serum phosphate levels and race and sex by including interaction terms in the models for the mean telomere length. Based on the statistically significant findings of the interaction effect, we used stratified test to perform further analyses.
